# Expression Quantitative Trait Locus Mapping Studies in Mid-secretory Phase Endometrial Cells Identifies *HLA-F* and *TAP2* as Fecundability-Associated Genes

**DOI:** 10.1371/journal.pgen.1005858

**Published:** 2016-07-22

**Authors:** Courtney K. Burrows, Gülüm Kosova, Catherine Herman, Kristen Patterson, Katherine E. Hartmann, Digna R. Velez Edwards, Mary D. Stephenson, Vincent J. Lynch, Carole Ober

**Affiliations:** 1 Department of Human Genetics, The University of Chicago, Chicago, Illinois, United States of America; 2 Institute for Medicine and Public Health, Vanderbilt Epidemiology Center, Vanderbilt University, Nashville, Tennessee, United States of America; 3 Departments of Obstetrics and Gynecology, Vanderbilt University Medical Center, Nashville, Tennessee, United States of America; 4 Department of Medicine, Vanderbilt University Medical Center, Nashville, Tennessee, United States of America; 5 Vanderbilt Genetics Institute, Vanderbilt University, Nashville, Tennessee, United States of America; 6 Department of Obstetrics and Gynecology, The University of Chicago, Chicago, Illinois, United States of America; Cincinnati Children's Hospital Medical Center, UNITED STATES

## Abstract

Fertility traits in humans are heritable, however, little is known about the genes that influence reproductive outcomes or the genetic variants that contribute to differences in these traits between individuals, particularly women. To address this gap in knowledge, we performed an unbiased genome-wide expression quantitative trait locus (eQTL) mapping study to identify common regulatory (expression) single nucleotide polymorphisms (eSNPs) in mid-secretory endometrium. We identified 423 cis-eQTLs for 132 genes that were significant at a false discovery rate (FDR) of 1%. After pruning for strong LD (*r*^2^ >0.95), we tested for associations between eSNPs and fecundability (the ability to get pregnant), measured as the length of the interval to pregnancy, in 117 women. Two eSNPs were associated with fecundability at a FDR of 5%; both were in the HLA region and were eQTLs for the *TAP2* gene (*P* = 1.3x10^-4^) and the *HLA-F* gene (*P* = 4.0x10^-4^), respectively. The effects of these SNPs on fecundability were replicated in an independent sample. The two eSNPs reside within or near regulatory elements in decidualized human endometrial stromal cells. Our study integrating eQTL mapping in a primary tissue with association studies of a related phenotype revealed novel genes and associated alleles with independent effects on fecundability, and identified a central role for two HLA region genes in human implantation success.

## Author Summary

Little is known about the genetics of female fertility. In this study, we addressed this gap in knowledge by first searching for genetic variants that regulate gene expression in uterine endometrial cells, and then testing those functional variants for associations with the length of time to pregnancy in fertile women. Two functional genetic variants were associated with time to pregnancy in women after correcting for multiple testing. Those variants were each associated with the expression of genes in the HLA region, *HLA-F* and *TAP2*, which are have not previously been implicated female fertility. The association between *HLA-F* and *TAP2* genotypes on the length of time to pregnancy was replicated in an independent cohort of women. Because *HLA-F* and *TAP2* are involved in immune processes, these results suggest their role in specific immune regulation in the endometrium during implantation. Future studies will characterize these molecules in the implantation process and their potential as drug targets for treatment of conditions related to implantation failure.

## Introduction

Natural variation in fertility traits is heritable in humans [1], yet identifying genes contributing to these traits remains challenging. Although genome-wide association studies (GWAS) have identified variants associated with many other complex phenotypes, its application to fertility traits is challenging. In particular, widespread contraceptive use among fertile couples and significant clinical heterogeneity among infertile couples makes it difficult to sample large numbers of fertile subjects with unprotected intercourse or infertile subjects whose inability to conceive results from the same underlying biological processes. Although a few GWAS of male fertility [2] or infertility [3, 4] traits have identified promising candidate genes, to date there have been no such studies in women.

To address these limitations, we have focused our genetic studies of fertility on members of a founder population, the Hutterites [1, 2, 5–8]. This communal living group of European ancestry has limited contraceptive use, a uniform desire for large families, a prohibition of smoking, and fertility rates that are among the highest ever reported [9, 10]. For example, only 2% of Hutterite couples are childless [9] compared to 10–15% of the general population [11]. Whereas miscarriages of clinically recognized pregnancies among Hutterite couples is 15.6% [8], nearly identical to estimates of clinically recognized miscarriage rates in outbred populations [12], recurrent miscarriages in childless couples are rare (0 of 525 interviewed Hutterite women [1]) compared to 5% in the general population [13]. Moreover, their communal lifestyle ensures that sociocultural factors influencing fertility are relatively uniform among Hutterite couples [1, 14]. We have proposed that their naturally high fertility rates, their reduced variance in environmental and lifestyle factors, and their limited gene pool due to the founder effect make the Hutterites an ideal population in which to dissect the genetic architecture of reproductive traits [1, 2, 15]. Here, we use an integrated strategy that first identifies a set of candidate regulatory single nucleotide polymorphisms (SNPs) in mid-secretory phase endometrium by expression quantitative trait locus (eQTL) mapping in non-Hutterite women with two or more previous miscarriages. We then tested for associations between those putatively functional expression (e)SNPs and fecundability in Hutterite women who are participants in a prospective study of pregnancy outcome [7, 8], and replicated the significant findings in an independent sample of women [16]. We report here the discovery of independent associations between SNPs that are eQTLs for the *HLA-F* and *TAP2* genes in mid-secretory phase endometrium and fecundability (the probability of achieving pregnancy), thereby implicating maternal HLA region genes for the first time in implantation processes.

## Results

### eQTL mapping in mid-secretory phase endometrium

We performed eQTL mapping in the mid-secretory phase endometrium, corresponding to the luteal phase of the ovarian cycle, from 53 women with two or more early pregnancy losses, using 378,362 common (≥10%) SNPs that were within 200kb of one or more of the 10,191 genes detected as expressed in these tissues (i.e., *cis*-eQTLs) (see Methods). We observed 423 cis-eQTLs (416 unique SNPs) for 132 genes at a false discovery rate (FDR) of 1% (S1 Dataset).

We next looked for gene ontology enrichments in the genes associated with eQTLs at a FDR of 1% using DAVID [17, 18] and GREAT [19]. We found an enrichment of the GO Biological Process of “antigen processing and presentation” (DAVID, FDR 2.5x10^-5^), the GO Molecular Function of “MHC class 1 receptor activity” (DAVID, FDR 5.30x10^-4^), and GO Cellular Component “MHC class 1 protein complex” (GREAT, FDR 2.02x10^-6^). Many of the DAVID and GREAT enrichments overlapped, and both highlighted the importance of immune related genes among those with eQTLs in mid-secretory phase endometrium.

To assess the clinical relevance of these eQTLs on female fertility traits, we first pruned the 416 SNPs for strong LD (*r*^2^ ≥ 0.95 in the Hutterites) and then carried forward 245 expression (e)SNPs for association studies in the prospective study participants.

### Association of eSNPs with fecundability in Hutterite women

We previously genotyped 208 of the 327 Hutterite women in a prospective study of pregnancy outcomes [6, 7, 15, 20] with the Affymetrix 500k or 6.0 genotyping chips. Using these genotypes as framework markers, we imputed all variants present in the whole genome sequences of 98 Hutterites to these women using PRIMAL, an imputation program that utilizes both pedigree- and LD-based imputation to provide on average of 87% coverage and >99% accuracy in the Hutterite pedigree [21]. From among the 245 (LD-pruned) SNPs that were eQTLs at a FDR 1%, genotypes for 189 (associated with the expression of 108 genes) were known for at least 85% of Hutterite women in the study of fecundability.

**Fig 1:**
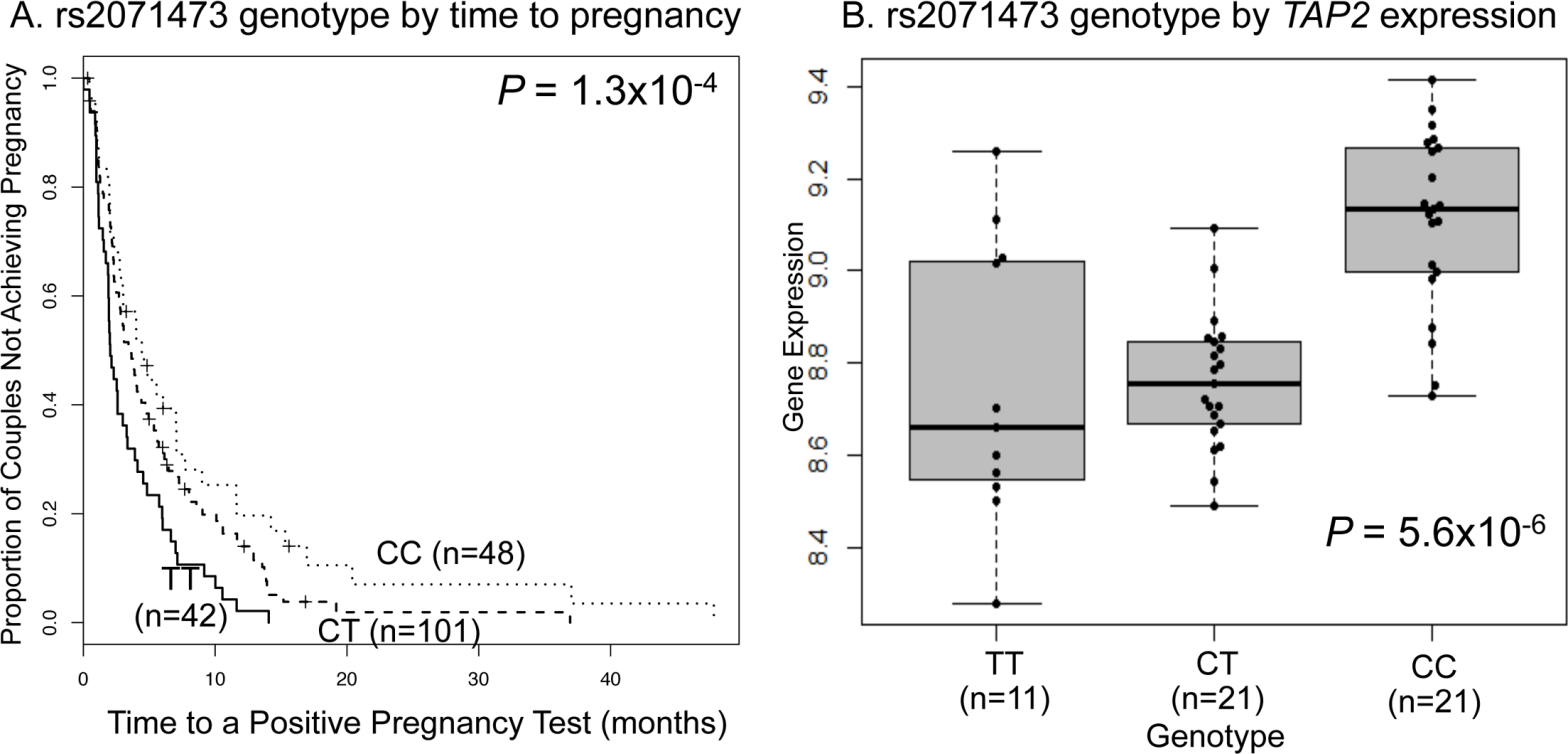
Associations between rs2071473 and fecundability and gene expression. **A.** Time-to-pregnancy curves by genotype in Hutterite women. The numbers in parentheses are the number of intervals included for women with each genotype. The number of women in each group is 28 (TT), 62 (CT) and 27 (CC). **B.** Expression levels of the *TAP2* gene in mid-secretory phase endometrial cells from women with recurrent early pregnancy loss. The numbers in parentheses are the number of intervals in each genotype group.

**Fig 2:**
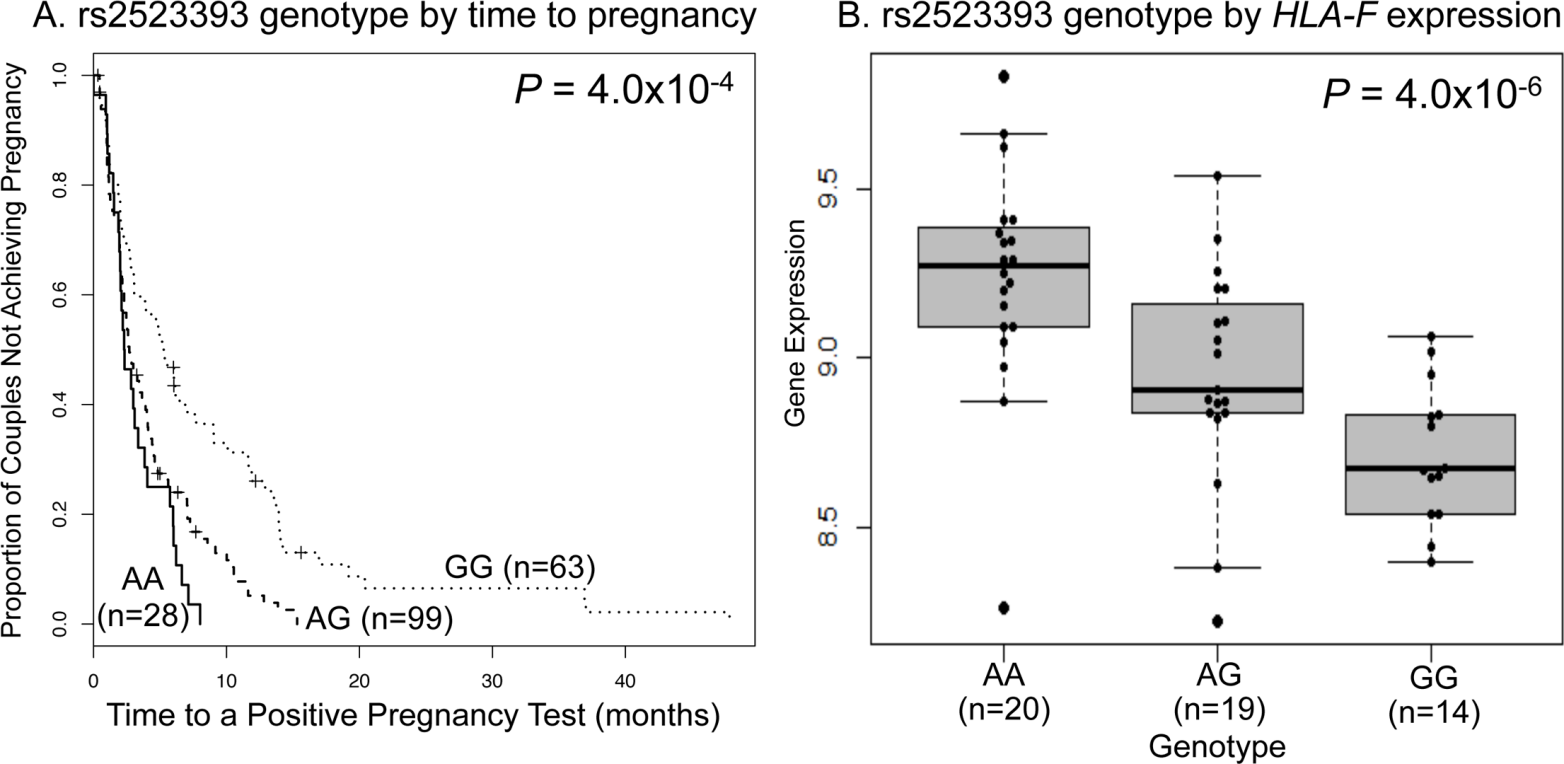
Associations between rs2523393 and fecundability and gene expression. **A.** Time-to-pregnancy curves by genotype in Hutterite women. The numbers in parentheses are the number of intervals included for women with each genotype. The number of women in each group is 16 (AA), 50 (AT) and 40 (GG). **B.** Expression levels of the *HLA-F* gene in mid-secretory phase endometrial cells from women with recurrent early pregnancy loss. The numbers in parentheses are the number of intervals in each genotype group.

We compared the length of the intervals from the resumption of menses after a prior pregnancy or miscarriage or following the discontinuation of birth control use (referred to as time0) to a positive pregnancy test in women who were not nursing at time0 of each included interval. For first pregnancies, we considered the length of the interval from the first menses after marriage to a positive pregnancy test (see Methods). If pregnancy occurred prior to the resumption of menses (or before the first period after marriage in first pregnancies), we considered the interval to be 14 days. Data were available for 191 intervals in 117 women (see Methods); 178 of the observed intervals resulted in a pregnancy at the time of the last follow-up. We used life-table analysis to compare intervals between genotype classes, adjusting for two significant covariates: maternal age and number of prior births (classified as 0–1, 2–3, or ≥4) (see Methods). Among the 189 eSNPs that we examined, genotypes for 21 were associated with the length of the interval to pregnancy at a *P* < 0.05 (Table 1). Two of these eSNPs were significant at a FDR of 5% and after Bonferroni correction for 189 tests. The most significant association was with rs2071473, an eSNP associated with expression of the *TAP2* gene in the HLA class II region; the C allele at this SNP was associated with longer intervals to pregnancy and higher expression of *TAP2* gene in mid-secretory phase endometrium (Fig 1). The median interval lengths to pregnancy were 2.0 (lower, upper quartile 1.2, 4.7), 3.1 (1.9, 6.2), and 4.0 (2.0, 7.6) months among women with the TT, CT, and CC genotypes, respectively, at this eSNP (*P* = 1.3x10^-4^). The second association was with rs2523393, an eSNP associated with expression of the *HLA-F* gene in the HLA class I region; the G allele at this SNP was associated with longer intervals to pregnancy and lower expression of *HLA-F* in mid-secretory phase endometrium (Fig 2). The median interval lengths to pregnancy were 2.3 (1.8, 4.5), 2.6 (1.4, 4.8), and 4.9 (2.0, 11.7) months among women with the AA, AG, and GG genotypes, respectively, at this eSNP (*P* = 4.0x10^-4^). For both eSNPs, intervals were longer and genotype differences more pronounced among women at lower parity (S1 and S2 Figs). Among the 21 eSNPs with *P* <0.05, nine (43%) were associated with expression of HLA region genes: one with *TAP1*, three with *HLA-F*, three with *HLA-G*, and two with *MICA*, consistent with the gene ontology analysis identifying enrichments for genes with antigen processing and presentation functions among those with eQTLs in mid-secretory phase endometrium. The results for all 189 eSNPs and their associated genes are shown in S2 Dataset.

**Table 1:** Fecundability and eQTL results for 21 eSNPs with p<0.05 in the analysis of fecundability. Results of the fecundability and eQTL analyses for all variants tested are presented in S1 and S2 Datasets, respectively. Base pair position is based on human genome build GRCh37/hg19. Variants are ordered by *P*-values in the fecundability analysis.

			Fecundability Analysis		eQTL Analysis		
rsID	Chr	Base Pair	*P*-Value	FDR	*P*-Value	FDR	Gene
**rs2071473**	**6**	**32782605**	**1.29x10^-4^**	**0.0243**	**5.62x10^-6^**	**0.0075**	***TAP2***
**rs2523393**	**6**	**29705659**	**3.97x10^-4^**	**0.0375**	**4.59x10^-6^**	**0.0065**	***HLA-F***
**rs2523405**	6	29695305	2.47x10^-3^	0.1301	7.86x10^-11^	0.0000	*HLA-F*
**rs16896724**	6	29919963	3.87x10^-3^	0.1301	4.07x10^-6^	0.0062	*HLA-G*
**rs6900894**	6	31387599	4.46x10^-3^	0.1301	2.34x10^-6^	0.0041	*MICA*
**rs11599615**	10	15395181	5.04x10^-3^	0.1301	3.09x10^-6^	0.0050	*FAM171A1*
**rs3869132**	6	31410948	5.64x10^-3^	0.1301	2.34x10^-6^	0.0041	*MICA*
**rs3787081**	20	25235129	6.05x10^-3^	0.1301	1.06x10^-6^	0.0023	*PYGB*
**rs12748456**	1	22370157	6.93x10^-3^	0.1301	6.58x10^-6^	0.0085	*LINC00339*
**rs1362126**	6	29691019	7.25x10^-3^	0.1301	2.18x10^-6^	0.0040	*HLA-F*
**rs11725932**	4	99799310	7.57x10^-3^	0.1301	2.11x10^-7^	0.0008	*EIF4E*
**rs2517930**	6	29745075	9.42x10^-3^	0.1483	2.74x10^-6^	0.0045	*HLA-G*
**rs7223165**	17	53363838	1.69x10^-2^	0.2455	1.65x10^-7^	0.0006	*HLF*
**rs12002855**	9	75435590	1.90x10^-2^	0.2570	3.00x10^-7^	0.0010	*ALDH1A1*
**rs9380145**	6	29802690	2.42x10^-2^	0.3046	1.78x10^-6^	0.0034	*HLA-G*
**rs28444847**	15	40356604	2.59x10^-2^	0.3063	4.80x10^-6^	0.0066	*SRP14*
**rs8036376**	15	39868627	3.10x10^-2^	0.3321	6.32x10^-6^	0.0082	*FSIP1*
**rs7229142**	18	677214	3.16x10^-2^	0.3321	1.02x10^-6^	0.0023	*ENOSF1*
**rs4239192**	17	53376504	3.75x10^-2^	0.3734	4.38x10^-7^	0.0013	*HLF*
**rs4808199**	19	19545099	4.07x10^-2^	0.3846	3.08x10^-7^	0.0010	*MAU2*
**rs5751939**	22	21139242	5.08x10^-2^	0.4572	1.59x10^-8^	0.0001	*SNAP29*

Previous studies of HLA and fertility in the Hutterites have shown that HLA matching between partners for alleles at the class II locus *HLA-DRB1* is associated with reduced fecundability, presumably due to the higher risk for class II compatible embryos among these couples [7]. To rule out that maternal-fetal compatibility at the *TAP2* or *HLA-F* locus accounts for the observed effects in this study we repeated the fecundability analysis, first stratifying couples based on husband’s genotype (rather than wife’s genotype) and then stratifying couples based on the wife’s genotype (as above) but now including the husband’s genotype as a covariate. We reasoned that if longer intervals are due to maternal-fetal compatibility and not maternal genotypes *per se*, then results of analyses stratifying by husband’s genotype should yield results similar to analyses stratified by wife’s genotype, and analyses including both husband’s and wife’s genotypes should be more significant than analyses considering either one individually. Neither eSNP was significant in the analysis considering the husband’s genotype as a main effect on length of intervals to pregnancy (*HLA-F* rs2523393 *P* = 0.94; *TAP2* rs2071473 *P* = 0.56). When husband’s genotype was included as a covariate in the model, the *P*-values were reduced from 4.0x10^-4^ to 0.0014 for rs2523393 and from 1.3x10^-4^ to 0.0015 for rs2071473, but the effect size associated with the risk alleles remained largely unchanged (β coefficients changed from 0.39 to 0.34 for rs2523393 and -0.44 to -0.36 rs2071473 when husbands’ genotypes were included as a covariate). These data indicate that maternal genotype at these two eSNPs is driving the association with time to pregnancy; there is no evidence for paternal or fetal genotype effects at these eSNPs contributing to interval lengths.

### Independent and combined effects of *TAP2* and *HLA-F* eSNPs on fecundability

**Fig 3:**
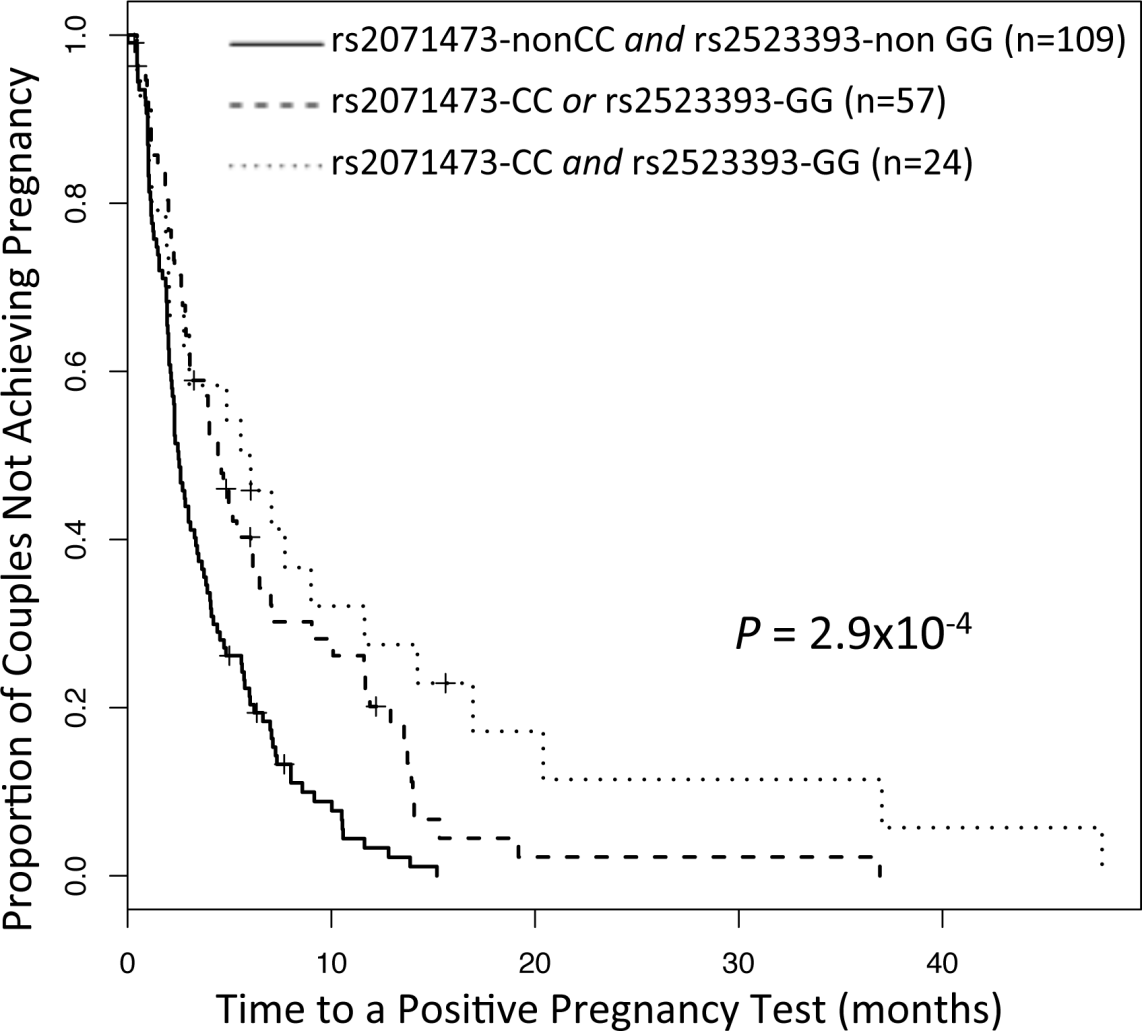
Combined analysis of rs2071473 and rs2523393 in the Hutterites. Time-to-pregnancy curves stratified by homozygosity for risk alleles at both eSNPs. The numbers in parentheses are the number of intervals included for women in each group. The number of women in each group is 64 (neither CC nor GG), 37 (CC or GG) and 15 (CC and GG).

Although these two eSNPs reside at opposite ends of the HLA region and are separated by ~3Mb, there are moderate levels of LD between them in the Hutterites (*r*^*2*^ *=* 0.19). To determine the statistical independence of the associations with fecundability, we repeated the time to pregnancy analysis but included the genotype at the other eSNP as a covariate. In the analysis of rs2071473 (*TAP2*) that included genotype at rs2523393 (*HLA-F*) as a covariate, the effect size and *P*-value changed from β = 0.39 (*P* = 1.3x10^-4^) to β = 0.23 (*P* = 0.0064); in the analysis of the rs2523393 (*HLA-F*) that included genotype at rs2071473 (*TAP2*) as a covariate, the effect size and *P*-value changed from β = -0.44 (*P* = 4.0x10^-4^) to -0.34 (*P* = 0.047). Thus, while the magnitude of each association is reduced in the analyses conditioning on the alternate eSNP, both retain independent effects on fecundability. The observed reduction in β values and significance is likely due to the LD between the SNPs. To further examine this, we stratified the women into three groups based on being homozygous at both, one, or neither of the high risk (longer interval) alleles at each eSNP (CC at rs2071473 [*TAP2*] and GG at rs2523393 [*HLA-F*]) (Fig 3). If the effects at these two loci were independent, then women who are homozygous for the high risk allele at both eSNPs should have longer intervals than women who are homozygous at only one or neither high risk allele. Indeed, intervals to pregnancy were longest among women homozygous for both rs2071473-CC *and* rs2523393-GG (median interval 5.2 months [1.9, 11.0 months]), intermediate among homozygous for only one of the high risk alleles (median interval 4.0 months [2.1, 9.3 months], and shortest among women who were not homozygous for either high risk allele (median interval 2.4 months [1.4, 4.8 months]) (*P* = 2.9x10^-4^). Moreover, women homozygous for the risk alleles at both the *TAP2* and *HLA-F* eSNPs had significantly longer intervals compared to women who were homozygous for a risk allele at only one of the two eSNPs (*P* = 1.6x10^-5^). Taken together these analyses indicate that the *TAP2* and *HLA-F* associations are independent and have additive effects on fecundability.

### Replication studies in the RFTS cohort

**Fig 4:**
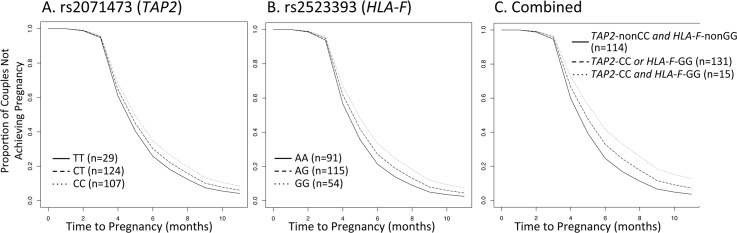
Replication of fecundability associated SNPs in RFTS. Time-to-pregnancy curves stratified by genotype. **A.** rs2071473 (*P* = 0.083), **B.** rs2523393 (*P =* 0.15), **C.** both SNPs (*P* = 0.033). The numbers in parentheses are the number of women in each genotype group (one interval per woman).

Using the same approach as that used in the Hutterites, we first examined the genotype effects of each SNP on fecundability and then the joint effects of the combined genotypes at each locus. At rs2071473, the *TAP2* eSNP, the median interval lengths to pregnancy were 5.0 (4.0, 7.0), 6.0 (4.0, 8.2), and 6.0 (4.0, 9.0) months among women with the TT, CT, and CC genotypes, respectively (*P* = 0.083; Fig 4A). At rs2523393, the *HLA-F* eSNP, the median interval lengths were 5.0 (4.0, 8.5), 6.0 (4.0, 8.0), and 6.0 (5.0, 10.0) months among women with the AA, AG, and GG genotypes, respectively (*P* = 0.155; Fig 4B). Although these results did reach nominal significance in the RFTS cohort, the 95% confidence intervals of the ORs in the Hutterites and RFTS cohort overlap (S1 Table). In the combined analysis, intervals to pregnancy were longest among women homozygous for both rs2071473-CC *and* rs2523393-GG (median interval 8.0 months [5.5, 12.5 months]), intermediate among homozygous for only one of the high risk alleles (median interval 6.0 months [4.0, 9.0 months], and shortest among women who were not homozygous for either high risk allele (median interval 5.0 months [4.0, 7.0 months]) (*P* = 0.033; Fig 4C), as we observed in the Hutterites.

### Fecundability-associated eSNPs are located within and near regulatory elements

Because there are many SNPs in strong LD with our lead eSNPs in the Hutterites, it cannot be inferred from association studies which of these linked SNPs are the true causal variants. To address this question, we used *in silico* analyses to determine which of the fecundability-associated eQTLs are in or near regulatory elements in decidualized human endometrial stromal cells [22, 23], ENCODE-annotated functional sites in the endometrial cell lines ECC-1 and Ishikawa [24], as well as the complete ENCODE regulatory element dataset. To interrogate more completely the variation in these regions, we used whole genome sequence data in the Hutterites to survey all variation in the 500kb windows flanking each of the two eSNPs that were associated with fecundability. After filtering our variants with minor allele frequencies <0.10 and call rates <85%, 4,442 variants remained in the *TAP2* region and 2,675 variants remained in the *HLA-F* region. We then filtered these variants based on their LD with each lead eSNP and retained the 70 variants with LD *r*^2^ ≥ 0.7 with rs2071473 and the 62 variants with LD *r*^2^ ≥ 0.7 with rs2523393. The variants that had LD *r*^2^ ≥ 0.7 with the lead eSNP in each region defined an approximately 6kb window around the lead SNP. We repeated the association studies with all variants within each 6kb window and fecundability. Because many of these variants were not included in the eQTL study in the 53 women with recurrent early pregnancy loss, we also imputed the missing genotypes using whole genome sequences from 100 European American individuals [25] (see Methods) and performed eQTL mapping in the mid-secretory phase endometrial RNA using these variants, as described above.

**Fig 5:**
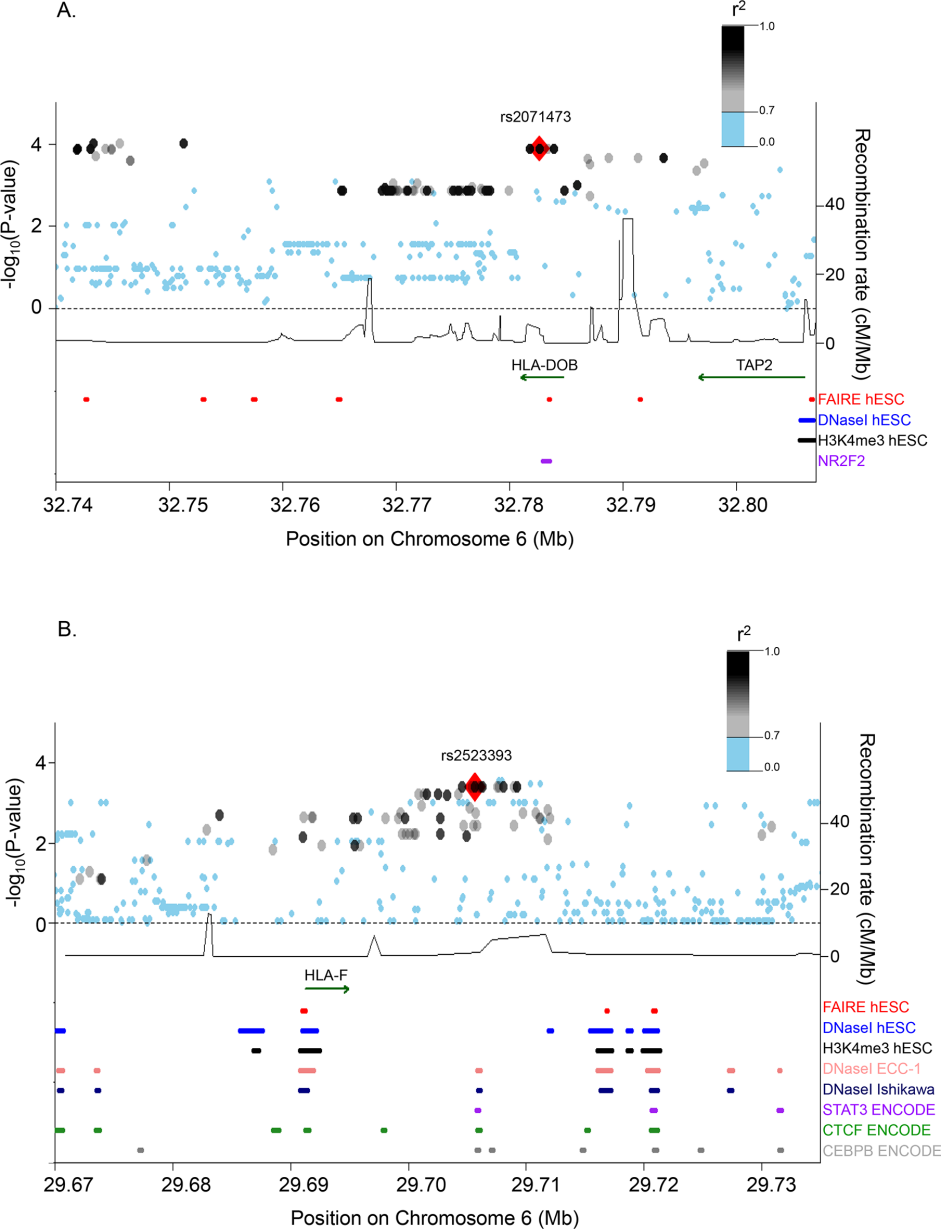
Regional associations with fecundability and functional annotations of the *TAP2* locus (Panel A) and the *HLA-F* locus (Panel B). The lead eSNP at each locus is shown as a red triangle; all other variants are color-coded based on their LD with the lead eSNPs. The upper section of each figure shows associations between variants within 6 kb of rs2071473 (A) or rs2523393 (B) and fecundability (left y-axis) and local recombination rates (right y-axis). The lower section of each figure shows the location of experimentally validated regulatory sites. Only genes that were detected as expressed in our eQTL study in mid-secretory endometrial cells are shown above the functional annotations. hESC, human endometrial stromal cells [23].

The lead eSNP at the *TAP2* locus, rs2071473, is located within an intron of the *HLA-DOB* gene and is near (~600bp) an NF2R2 transcription factor (TF) binding site in hESC; EP300, FOXM1, ATF2, and RUNX3 binding sites in a B-lymphoblastoid cell line (GM12878); and a DNase-I hypersensitivity site in 40 ENCODE cell lines (Fig 5A). This SNP is also within ~800bp of a FAIRE peak in hESC. Another SNP, rs2856995, that is 732bp upstream of and in perfect LD (*r*^2^ = 1) with rs2071473 resides within the NF2R2 binding site and 92bp upstream of the FAIRE site in hESC. Multiple other variants are located adjacent to FAIRE sites and among the 36 of these variants with eQTLs, 34 were eQTLs only for *TAP2* (FDR <15%), and two were eQTLs for *TAP1* (FDR = 14%) (S3 Dataset).

The lead eSNP at the *HLA-F* locus, rs2523393, is located within an intron of *HLA-F-AS1*, an antisense transcript that was not expressed in mid-secretory endometrium. The eSNP is about 10kb downstream of *HLA-F*, and in perfect LD (*r*^*2*^ = 1) with a cluster of SNPs ~360bp away that reside within multiple ENCODE-annotated functional sites in endometrial cell lines [26] (Fig 5B). One such SNP, rs2523389, is in a DNaseI hypersensitivity site in 120 ENCODE cell lines including the endometrial derived cell lines ECC-1 and Ishikawa treated with 10nM estradiol. This variant is also in a CTCF binding site that is present in 97 ENCODE cell lines, including the endometrial cell line ECC-1, and in a c-Myc binding site in a leukemia cell line. Another variant, rs2523391, is in a STAT3 binding site in a mammary gland cell line and 6bp from a CEBPB TF ChIP-seq binding site in HeLa and HepG2 cell lines, in addition to the same functional sites as rs2523389. Although the c-Myc, STAT3, and CEBPB binding sites were not present in the hESC or ENCODE endometrial cell lines, these transcription factors are essential for decidualization of endometrial stromal cells and the successful establishment of pregnancy [27–29]. Among the variants in LD with rs2523393 at *r*^2^ ≥ 0.7 and with eQTL results, all were most strongly associated with expression of *HLA-F* (S4 Dataset). One eSNP in the *HLA-F* promoter (rs1362126; *r*^*2*^ = 0.78 to rs2523393) was also an eQTL for *HLA-G* (FDR <1%), although to a lesser degree than for *HLA-F* (*HLA-F* eQTL *P* = 2.18x10^-6^, *HLA-G* eQTL *P* = 2.06x10^-3^).

Overall, these data indicate that our lead eSNPs and/or a small number of variants in perfect LD with those eSNPs are plausible causal candidates for the observed associations with fecundability in each region.

## Discussion

The mechanisms that allow the fetal allograft to avoid maternal immunologic rejection and survive over relatively long gestational periods in placental mammals are still incompletely known, although our understanding of these processes have advanced considerably since Medawar proposed this paradox over 60 years ago [30]. In particular, it has become clear that major histocompatibility complex (MHC) antigens, which play a central role in the rejection of non-self tissues, also contribute to maternal tolerance of the fetus, which is maintained in normal pregnancies. For example, our group previously demonstrated that matching of HLA antigens (the human MHC loci) between Hutterite couples is associated with longer intervals from marriage to each birth compared to couples not matching for HLA [31], and that longer intervals resulted from both higher miscarriage rates among couples matching for class I HLA-B antigens [8] and longer intervals to pregnancy among couples matching for class II HLA-DR antigens [7]. More recently, we reported associations between maternal *HLA-G* genotypes and miscarriage [6]. We and others have also shown associations between maternal or fetal *HLA-G* genotypes with recurrent pregnancy loss and preeclampsia [32–42]. Finally, recent studies have elegantly demonstrated that two HLA that are expressed by fetal extravillous cytotrophoblast (EV-CTB) cells at the maternal-fetal interface, HLA-G and HLA-C, are ligands for inhibitory receptors ILT2/IL4 and KIR2DL3, respectively, on immune cells [43–45]. Collectively these data indicate that multiple HLA molecules play important independent roles at the maternal-fetal interface in human pregnancy and that their effects can influence pregnancy outcome throughout gestation.

In this study, we hypothesized that perturbations of genes expressed in mid-secretory phase endometrial cells could affect implantation and be visible as delayed time to pregnancy in otherwise fertile couples. Although our study was unbiased with respect to genome location because we interrogated variation that was first identified through a genome-wide eQTL study, the results of both the eQTL study and the subsequent study of fecundability highlighted the importance of HLA region genes in achieving pregnancy. Among the eQTLs taken forward to studies of fecundability in the Hutterites, nine (43%) of the eSNPs with association *P*-values <0.05 were eQTLs for HLA region genes compared to 15% of all 189 eSNPs tested. Two of the associations with fecundability were significant at a FDR of 5%, remained significant after correction for multiple testing, and were replicated in an independent sample of fertile women: one SNP was with an eQTL for *TAP2* and one for *HLA-F*. To our knowledge neither of these two HLA region genes has previously been directly implicated in pregnancy processes. We further demonstrated that eQTLs for these two HLA loci in mid-secretory phase endometrium are independently associated with fecundability in fertile women and that neither paternal nor fetal genotype at these loci contributed to these effects. These findings may be particularly relevant to women with primary infertility of unknown etiology, with recurrent implantation failure following in vitro fertilization (IVF), or possibly even with recurrent early pregnancy loss.

Both genes are intriguing candidates for fecundability genes. The *TAP2* gene in the class II HLA region encodes the antigen peptide transporter 2 protein. TAP2 forms a heterodimer with TAP1 (encoded by the *TAP1* gene, located 7kb away) in order to transport peptides from the cytoplasm to the endoplasmic reticulum, where they are loaded into assembling class I HLA molecules prior to their transport to the cell surface. The association of TAP complex with HLA class I molecules, including HLA-F [46], is critical for their expression on the cell surface [47]. *HLA-F*, which is located ~3Mb telomeric to *TAP2*, encodes a class I HLA protein that is considered “non-classical” because it has limited coding polymorphisms and restricted tissue distribution [48], and functions that are still poorly characterized but likely distinct from the classical class I HLA (HLA-A, HLA-B, HLA-C). In fact, recent studies have shown that HLA-F physically interacts with the KIR3DL2 and KIR2DS4 receptors on natural killer (NK) cells [49], an abundant and critical cell in the maternal uterus that proliferates during the secretory phase and then throughout pregnancy [50]. Later in pregnancy, HLA-F is expressed in EV-CTB [51–54], although its function in placental cells is not well characterized [52]. Our combined results suggest that perturbations in expression of either gene in endometrial cells in the mid-secretory phase influences implantation success, with overexpression of *TAP2* and underexpression of *HLA-F* resulting in delayed time to pregnancy.

We found multiple variants in perfect LD with both eSNPs that reside in transcription factor binding sites and other regulatory elements in endometrial cell lines. The *TAP2*-associated variants are located within a NR2F2 (COUP-TFII) binding site. Multiple studies have shown that female mice deficient in NR2F2 have implantation failure, with impairments of both embryo attachment and uterine decidualization [55–57], and NR2F2 knock downs a human endometrial stromal cell line significantly reduces TAP2 expression [22]. These combined data suggest a potential mechanism for the association we observed with expression of *TAP2* and fecundability in women. At the *HLA-F* locus, variants associated with gene expression level and fecundability are in DNaseI hypersensitivity sites, a marker of open chromatin and transcriptional activity, in multiple human endometrial cell lines [26], suggesting that one or more of these variants may indeed be causally associated with both gene expression and fecundability.

Although genome-wide association studies can be powerful approaches for identifying susceptibility loci for common diseases and complex phenotypes, they require very large sample sizes that may be infeasible to acquire for many important phenotypes. We used an alternative approach for mapping fecundability genes by first reducing the search space to SNPs that were associated with gene expression in a relevant tissue and then taking this smaller set of regulatory SNPs forward to an association study in carefully phenotyped subjects. This approach revealed two novel associations with fecundability and immediate intuition regarding the genes underlying each association, the relationship between gene expression and fecundability, and potential mechanisms for these associations. Future studies will be required to characterize the role of these molecules in the implantation process and to evaluate their potential as drug targets for treatment of conditions related to suboptimal implantation.

## Materials and Methods

### Study subjects and sample collection for eQTL mapping studies

Fifty-eight women underwent endometrial biopsies as part of their clinical evaluation for recurrent pregnancy loss at the University of Chicago, after obtaining informed consent. These women were between the ages of 26 and 43 years and had at least two previous pregnancy losses before10 weeks gestation. Fifty-two (90%) were of European ancestry, two (3%) were of Asian ancestry, and four (7%) were of African ancestry. Medical records and individual diagnoses were not available to us for this study, and all women were included in the expression studies. Because recurrent miscarriage can result from many potential causes, and nearly half remain unexplained after evaluation, we reasoned that gene expression in the endometrium from these women would maximize variation in gene expression and increase our power to detect eQTLs. We were unable to obtain samples from women without a history of pregnancy loss for this study.

Endometrial biopsies were performed during the mid-secretory phase (9–11 days after endogenous luteinizing hormone [LH] surge, detected by each woman testing her daily urine) and immediately frozen on dry ice; samples were stored at -80°C until RNA was extracted, as previously described [58]. Histological examination of the biopsies confirmed endometrial tissue from the fundus of the uterus; endometrial glands and epithelium were present.

### Gene expression studies

RNA was extracted from the endometrial biopsies using a phenol-chloroform phase separation with TRIzol per the manufacturer’s directions (Life Technologies Corp., Carlsbad, CA, USA) and RNeasy RNA extraction kit (Qiagen, Venlo, Netherlands; per manufacturer’s directions). RNA quality was assessed using the Agilent 2100 Bioanalyzer (Agilent Technologies, Santa Clara, CA). The average RNA integrity number (RIN) score [59] was 8.06 (range 6.7–9.3). We were unable to obtain RIN scores for two samples due to extremely low concentration. However, these two samples passed all gene expression QC and were therefore included in the eQTL mapping studies. Gene expression was measured in RNA from 58 individuals. We included triplicate samples for three women and duplicate samples for 29. Gene expression was interrogated using the Human HT12v4 Expression BeadChip (Illumina, Inc., San Diego, CA, USA), which contains 47,231 probes that target 11,121 unique RefSeq genes. cDNA synthesis, hybridization, scanning and image processing and returned probe intensity measurements were performed at the University of Chicago Functional Genomics Core. Intensity estimates were log-transformed and quantile normalized using the ‘lumi’ package in R [60] (see S3 Fig). To remove probes for targets that were likely not expressed, all probes that did not have a detection P-value <0.05 in at least 70% of the samples were removed (leaving 17,208 probes). We further removed probes that did not map uniquely to the HG19 genome using Burrows-Wheeler Aligner (BWA), and probes that contained CEU HapMap SNPs with the QC+ designation (4,825 probes total excluded). After quality control (QC), 12,383 probes remained and were included in our eQTL studies. Probe averages were taken for replicate samples. Of the 11,121 unique genes targeted on the array, 2,192 (20%) had multiple probes [61]. For those genes with multiple probes, we chose the most 3’ probe in the gene to estimate expression. Samples from two women were excluded prior to QC because there were too few probes detected after hybridization (13,189 and 15,863 probes, respectively, compared to a median count of 21,341 out of 47,231 probes on the array); and three women were excluded prior to analysis because there was no DNA available for one and low genotyping call rates in two (see below). The remaining 53 women (49 European ancestry, 2 Asian ancestry, 2 African American ancestry) had both high quality expression and genotype data. In those 53 samples, 10,191 genes were detected as expressed. Processing batch and array were significantly associated with the variance in gene expression based on principle component (PC) analysis and their effects were regressed out using a linear model (see S4 Fig). Other covariates that were considered (age, BMI, race, and season of biopsy collection) were not significant in these samples (see S2 Table). An overview of the sample inclusion pipeline is shown in S5A Fig.

### Genotyping

DNA from women in the gene expression studies were genotyped with the Affymetrix Axiom Genome-Wide CEU 1 Array at the UCSF Genomics Core Facility. We performed QC checks using PLINK [62], and removed 4,922 SNPs with <95% genotype call rates, 503 with Hardy-Weinberg *P*-values ≤0.001, 336 non-autosomal SNPs, and 252,872 with minor allele frequencies <0.10. There were 370,008 SNPs remaining. After excluding two women with low call rates, the remaining 53 subjects had SNP call rates >97%.

### eQTL mapping

Linear regression was used to test for associations between the expression levels of 10,191 genes and genotypes at the 378,362 SNPs with a minor allele frequency greater than 10%, using the R package Matrix eQTL [63]. Genotypes were recoded as 0, 1, or 2 to reflect an additive model. To maximize the power in our sample, we tested for associations only with SNPs within ~200kb of the transcription start site of a gene, a distance that would include nearly all cis regulatory SNPs [64–66]. The Matrix eQTL package assigns both a p-value and a false discovery rate (FDR) to each SNP-gene association. The FDR was calculated using the Benjamini and Hochberg [67] procedure (see [63] for detailed methods). eQTL mapping was additionally performed including only the 49 women of European ancestry. As expected, p-values were generally less significant in the smaller sample, however the *HLA-F* and *TAP2* eQTLs remained significant at a FDR <1% (S5 Dataset).

### Pathway analysis

We used the Database for Annotation, Visualization and Integrated Discovery (DAVID) v6.7 [17, 68] to interrogate pathway and gene enrichment for eQTLs at a FDR 1% compared to all gene-SNP combinations in our analysis (background). An enrichment score was calculated using Fisher's Exact test (modified as EASE score) on gene count for eQTLs at a FDR of 1% compared to all genes tested. We used the high classification stringency for our analysis.

We also used the Genomic Regions Enrichment of Annotations Tool (GREAT) to analyze the significance of SNPs which are eQTLs at a FDR of 1% [19]. GREAT first associates genomic regions with nearby genes and then applies the functional annotations for those genes to the regions. We used the basal plus extension definition of a gene regulatory domain, in which a gene’s defined regulatory domain expands until it reaches the nearest gene’s basal domain or maximally 5kb upstream and 1kb downstream. Using this definition, SNPs located between two genes may include both gene regulatory domains. GREAT uses a hypergeometric test over these defined genomic domains to assess enrichment between foreground (FDR 1% eQTLs) and background (all SNPs for which there is a gene-SNP pair tested in the eQTL analysis).

### Prospective study of pregnancy outcome in Hutterite women

The data included in our analyses of fecundability were derived from a prospective study of pregnancy outcome in South Dakota Hutterites that was initiated in 1986, as previously described [6–8, 69]. The women in this study are provided with calendar diaries and EPT pregnancy test kits (Warner-Lambert Co.). They record in the diary dates of menses, changes in nursing patterns, illnesses or travel for the husband and wife, and dates of miscarriages or deliveries. In addition, they are instructed to test for pregnancy if they do not start menses exactly one month after the first day of their previous period, and to record the results of all pregnancy tests in the diaries. They are also asked to start testing for pregnancy on a monthly basis starting 6 months after delivery until menses resumes. Results of all pregnancy tests and outcomes of each pregnancy are recorded in the diaries, which are collected yearly either in person or through the mail. The results reported here include data collected through 2013.

### Fecundability analysis

Among the 325 Hutterite women who have participated in the prospective study, 156 women had at least one interval during which they were not nursing at time0. Of these 156 women, genotype data were available for 117. The following studies were performed in these 117 women, who provided information on 191 intervals. An overview of the sample inclusion pipeline is shown in S5B Fig.

The distribution of the interval lengths until a positive pregnancy test was compared between genotype groups using non-parametric life-table analyses [70]. The product-moment method was used to compute the time-to-pregnancy curves. These curves were compared with the Mantel-Haenszel log-rank. Women were stratified based on their genotype. Potential confounding effects of maternal age at the beginning of each interval, number of prior miscarriages, number of births (parity), maternal birth year, maternal inbreeding, and the kinship coefficient of the couple were assess by Cox regression analysis, as previously described [7]. Only maternal age and number of prior births (parity) were significant covariates and included in subsequent analyses. Because the Cox model assumes that the hazard ratios for continuous variables are log linear, we classified women (at each observation) by the number of prior births in three categories: 0–1, 2–3, or ≥4 prior births.

### Replication studies in *Right from the Start (RFTS)*

RFTS is a pregnancy cohort that enrolled study participants from the community between 2001 and 2012. Participants were recruited from Galveston, Texas; Memphis, Nashville, Knoxville, and Chattanooga, Tennessee; and the Research Triangle region (Raleigh, Durham, and Chapel Hill) in North Carolina. Detailed descriptions of direct marketing and recruitment strategies have been previously described [16].

Participants completed a baseline interview at enrollment and a computer assisted telephone interview at the end of the first trimester. All pregnancies were confirmed by pregnancy tests performed either by their provider (with confirmation), the study staff, or by the participant with BFP Early Pregnancy Test Strips (Fairhaven Health) provided by the study staff. The baseline and first trimester interviews provided information on reproductive history and potential confounders. Information about fecundability was collected on the baseline interview. Women were asked to report the number of cycles or months that it took them to conceive (if pregnant) or how long they have been trying to become pregnant (if not pregnant). Time to pregnancy was censored after 11 months; women in the study did not use any contraception during this time. We considered only one interval (pregnancy) per woman. DNA samples were obtained either in person or by mail during follow-up using Oragene saliva DNA kits (DNA Genotek Inc., Ontario, Canada).

These analyses included 314 RFTS participants who were 18 years or older, non-Hispanic white, and had self-reported time-to-pregnancy. In these women, we genotyped the two SNPs associated with fecundability in the Hutterites (rs2071473 and rs2523392) using TaqMan assays. We then excluded from subsequent analyses 22 women who did not know or declined to answer questions about their contraceptive practices, eight women who could not provide information on their cycle length, 22 women whose cycle lengths were <21 days or >35 days, one woman with cycle lengths >35 days and did not respond to questions about her contraceptive practices, and one woman with missing genotype data. These exclusions resulted in 260 women that were included in subsequent analyses. An overview of the sample inclusion pipeline is shown in S5C Fig.

The genotypic effects on time to pregnancy were estimated using a discrete time hazard model, a discrete time analog to the Cox proportional hazards model. We considered as covariates maternal age, education, marital status, income, smoking, alcohol use, caffeine consumption, body mass index, number of previous pregnancies, number of previous elective pregnancy terminations, and whether they were pregnant at the start of the study using a forward selection method. Number of previous elective terminations (range 0–2; median = 0) was inversely associated with interval lengths (*P* = 0.028) and being pregnant at the start of the study was associated with shorter interval lengths (*P* = 0.0016). These two covariates were included in the analysis of genotype effects. Although age and number of previous pregnancies (parity groups: 0–1, 2–3, ≥4) were not significant predictors of interval lengths in the RFTS study (maternal age, *P* = 0.91; parity groups compared to 0–1, *P* = 0.26 and 0.74, respectively), we included them in the model to be consistent with the analysis in the Hutterites.

### Genotype imputation for eQTL mapping

Variants that were present in the Hutterites in each of the two associated regions but were not genotyped in the women with recurrent pregnancy loss who were included in the eQTL study were imputed in those women for a second stage eQTL mapping. We used whole genome sequences from 100 European American individuals as the reference genotypes for imputation [25]. Before imputation, variants with minor allele frequencies <1% or genotype call rates <95% were removed and variants on the reverse stand were flipped to the forward strand using PLINK [62]. To decrease computation time, we pre-phased the haplotypes in the reference genomes using Mach [71]. We then imputed genotypes in the women in the eQTL study using Minimac [72]. To avoid using SNPs with low imputation quality, we removed SNPs with an estimated R^2^ less than 0.5 before performing the eQTL mapping. We were able to impute or directly genotype 117 of the 7,062 variants in the two associated regions.

### Ethics statement

The eQTL mapping study in women with recurrent pregnancy loss was approved by the University of Chicago IRB (protocol number 14599B); all women gave written consent. The prospective study in the Hutterites was approved by the University of Chicago IRB (protocol number 5444); all participants gave written consent. The RFTS study was approved by Vanderbilt Human Research Protection Program (VHRPP) and Vanderbilt IRB (protocol numbers 070037 and 100396); all participants gave both verbal and written consent.

## Supporting Information

S1 Fig Associations between genotype at rs2071473 and fecundability by parity. Time-to-pregnancy curves by genotype and parity (previous pregnancies) in Hutterite women. The numbers in parentheses in the legend and in the numbers in the table on the right are the number of intervals (observations), number of completed pregnancies, and number of women within each genotype and/or parity strata. In the figure, results by genotype have been stratified by number of previous pregnancies (0–1, 2–3, ≥4). MAs = minor alleles, PPs = Previous pregnancies
(PDF)

S2 Fig Associations between genotype at rs2523393 and fecundability by parity. Time-to-pregnancy curves by genotype and parity (previous pregnancies) in Hutterite women. The numbers in parentheses in the legend and in the numbers in the table on the right are the number of intervals (observations), number of completed pregnancies, and number of women within each genotype and/or parity strata. In the figure, results by genotype have been stratified by number of previous pregnancies (0–1, 2–3, ≥4). MAs = minor alleles, PPs = Previous pregnancies
(PDF)

S3 Fig Boxplots of sample microarray intensity before and after quantile normalization. Boxplots of sample microarray intensity before (Panel A) and after (Panel B) quantile normalization.
(TIF)

S4 Fig Principal components (PC) analysis to interrogate the effects of covariates on gene expression data. The PC plots show each sample (before replicates were averaged) and is colored coded by the batch, which corresponds here to preparation of samples by two separate technicians and days of hybridization. Batch one includes chips one and two and batch two includes chips three through nine. The top panels (A and B) show PCs one through three for all samples before regressing out batch and chip. The bottom panels (C and D) shows the same PCs after regressing out batch and chip.
(TIF)

S5 Fig Overview of sample inclusion pipeline. Panel A shows the inclusion scheme for women in the eQTL analysis. Panel B shows the inclusion scheme for women in the fecundability analysis. Lastly, Panel C shows the inclusion scheme for women in the Right from the Start (RFTS) Study Analysis.
(TIF)

S1 Dataset 423 cis-eQTLs for 132 genes at a FDR of 1%. (XLSX)

S2 Dataset 189 eSNPs and results with fecundability and eQTL analysis. (XLSX)

S3 Dataset *TAP2* region fecundability and eQTL analysis. (XLSX)

S4 Dataset *HLA-F* region fecundability and eQTL analysis. (XLSX)

S5 Dataset 315 cis-eQTLs for 111 genes at a FDR of 1% in women with European ancestry. (XLSX)

S1 Table Comparison of genotype effect sizes, odds ratios, and 95% confidence intervals in the Hutterite and RFTS samples. The Cox proportional hazards model was used in the Hutterites because intervals were measured as the difference between two dates (continuous variable), whereas the discrete time hazards model was used in the RFTS cohort because intervals were measured as number of months (discrete variable).
(XLSX)

S2 Table Analysis of covariate effects on PCs. P-values correspond to the significance of each covariate in a linear model with each PC of expression data. Batch and chip were significant associated with PC1 through PC4, and number of previous miscarriages with PC2 (Bonferroni-adjusted p-value = 0.007). After regressing out the effects of batch and chip, none of the covariates are significantly associated with a PC. Season was defined as four three-month categories.
(XLSX)
