# Supplementary material for: Expression Quantitative Trait Locus Mapping Studies in Mid-secretory Phase Endometrial Cells Identifies HLA-F and TAP2 as Fecundability-Associated Genes
Source: PLoS Genet. 2016 Jul 22;12(7):e1005858. doi: 10.1371/journal.pgen.1005858 (PMC4957750; doi:10.1371/journal.pgen.1005858)
Supplement: S1 Fig — Time-to-pregnancy curves by genotype and parity (previous pregnancies) in Hutterite women. The numbers in parentheses in the legend and in the numbers in the table on the right are the number of intervals (observations), number of completed pregnancies, and number of women within each genotype and/or parity strata. In the figure, results by genotype have been stratified by number of previous pregnancies (0–1, 2–3, ≥4). MAs = minor alleles, PPs = Previous pregnancies (PDF) [file pgen.1005858.s001.pdf]

# Time To Pregnancy Curves for rs2071473 (eQTL for *TAP2*)

(n=#observations, # pregnancies, # women)

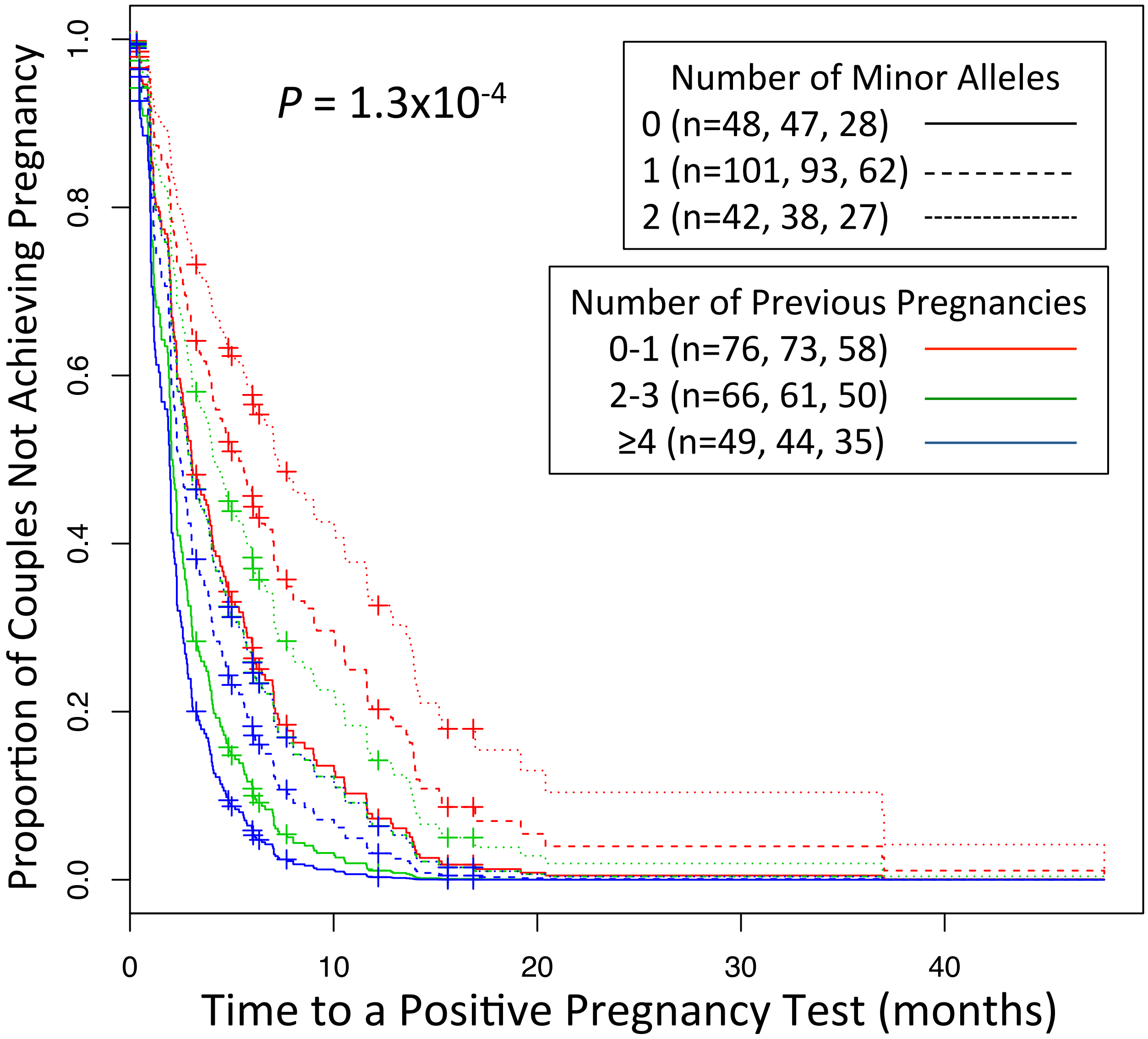


**Sample Sizes by Stratum**

| # MAs | # PPs | n |
|---|---|---|
| 0 | 0-1 | 17, 17, 14 |
| 0 | 2-3 | 15, 14, 11 |
| 0 | ≥4 | 16, 16, 11 |
| 1 | 0-1 | 38, 36, 30 |
| 1 | 2-3 | 38, 34, 29 |
| 1 | ≥4 | 25, 23, 17 |
| 2 | 0-1 | 21, 20, 14 |
| 2 | 2-3 | 13, 13, 10 |
| 2 | ≥4 | 8, 5, 7 |
