# Supplementary figures and images for: Expression Quantitative Trait Locus Mapping Studies in Mid-secretory Phase Endometrial Cells Identifies HLA-F and TAP2 as Fecundability-Associated Genes
Source: PLoS Genet. 2016 Jul 22;12(7):e1005858. doi: 10.1371/journal.pgen.1005858 (PMC4957750; doi:10.1371/journal.pgen.1005858)

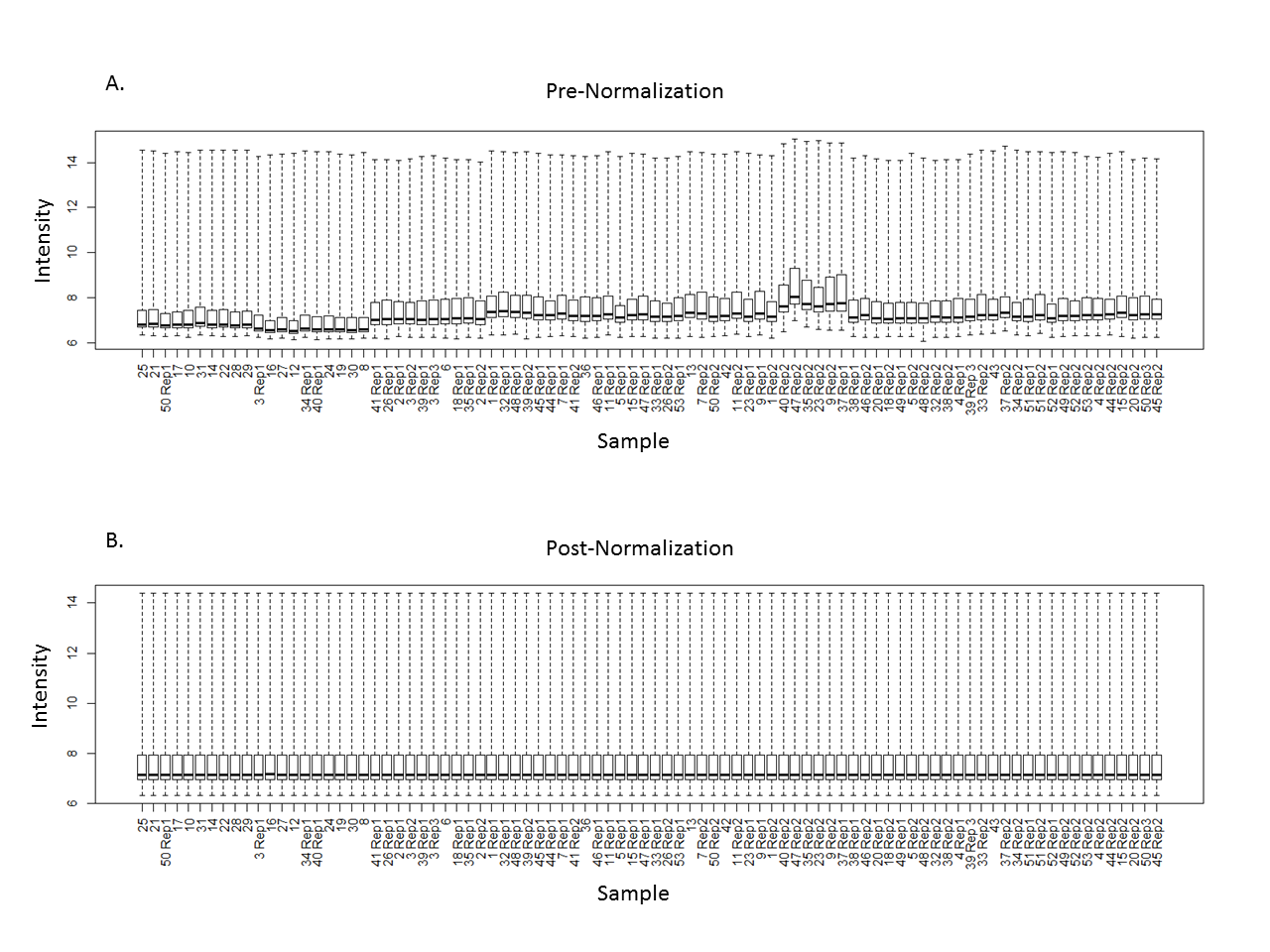

Supplement: S3 Fig — Boxplots of sample microarray intensity before (Panel A) and after (Panel B) quantile normalization. (TIF) [file pgen.1005858.s003.tif]

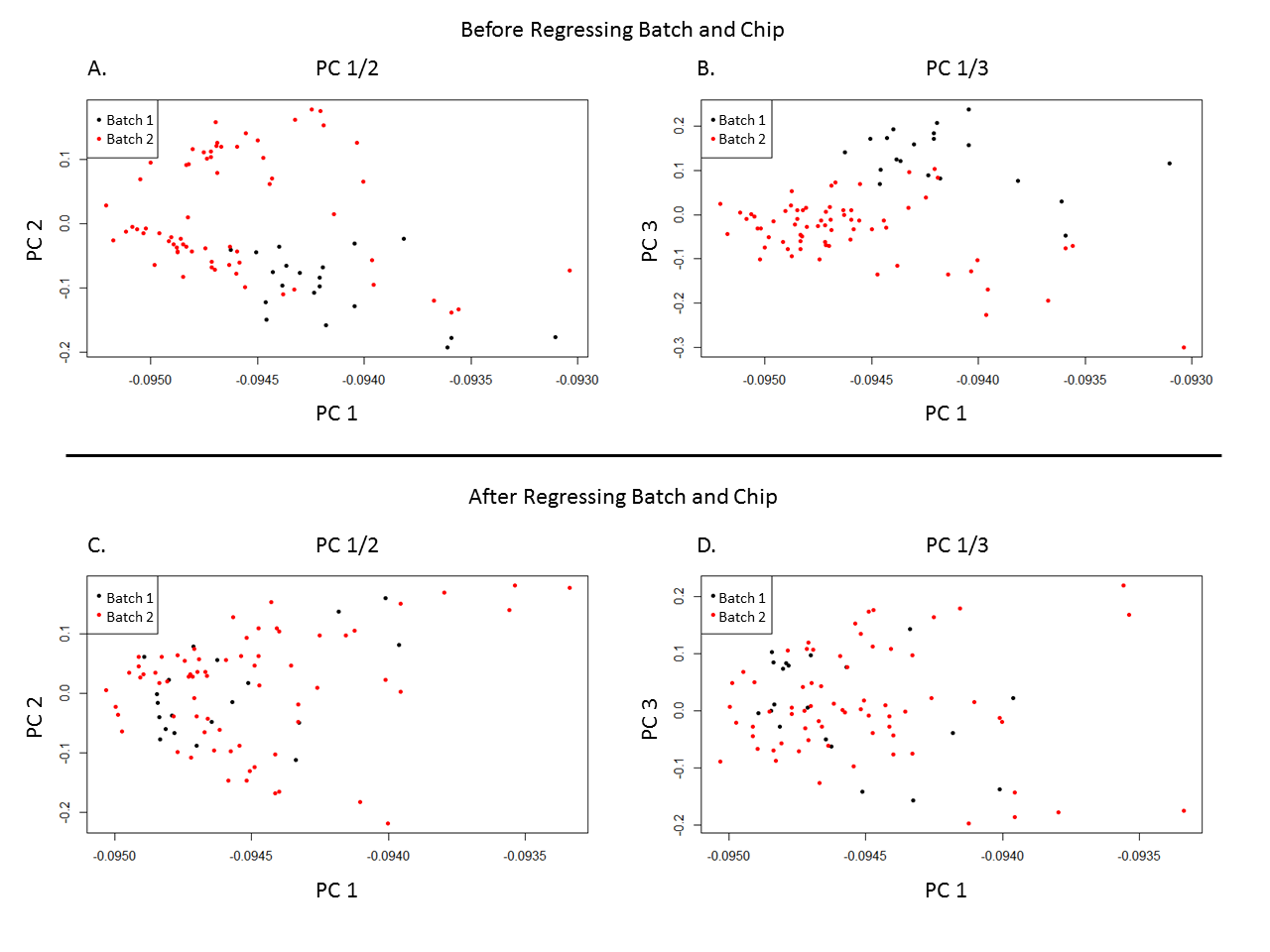

Supplement: S4 Fig — The PC plots show each sample (before replicates were averaged) and is colored coded by the batch, which corresponds here to preparation of samples by two separate technicians and days of hybridization. Batch one includes chips one and two and batch two includes chips three through nine. The top panels (A and B) show PCs one through three for all samples before regressing out batch and chip. The bottom panels (C and D) shows the same PCs after regressing out batch and chip. (TIF) [file pgen.1005858.s004.tif]

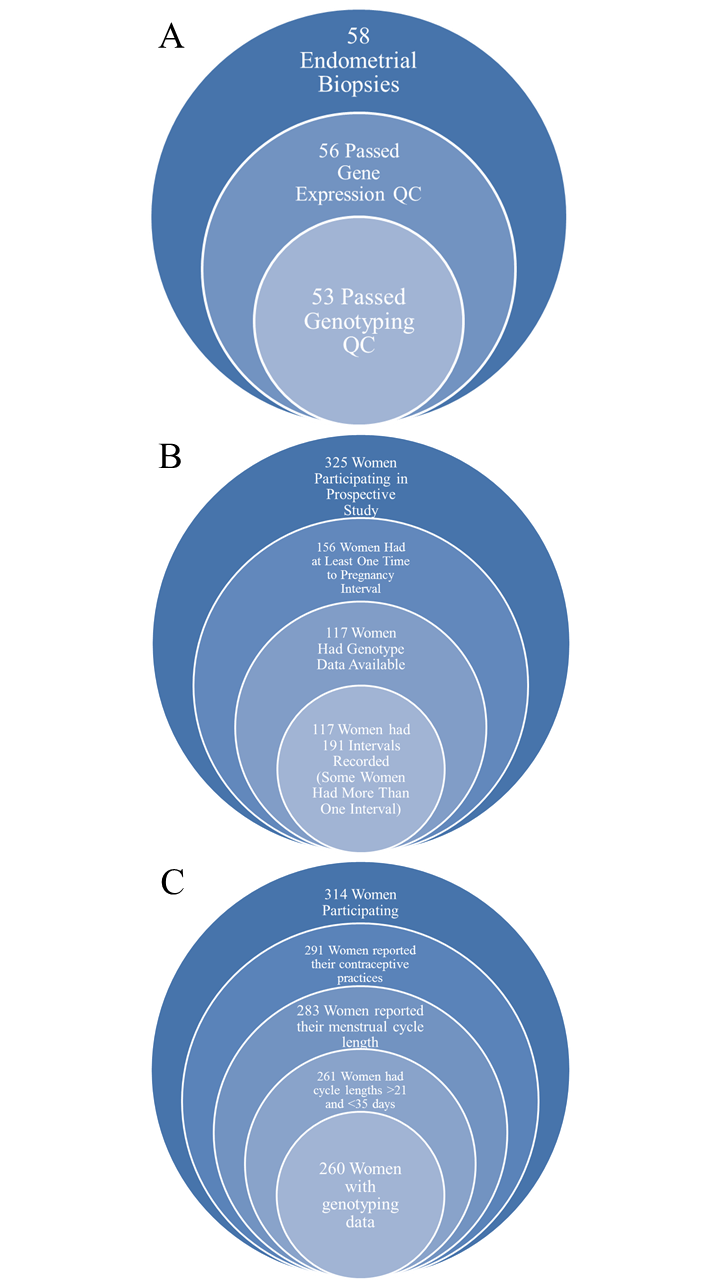

Supplement: S5 Fig — Panel A shows the inclusion scheme for women in the eQTL analysis. Panel B shows the inclusion scheme for women in the fecundability analysis. Lastly, Panel C shows the inclusion scheme for women in the Right from the Start (RFTS) Study Analysis. (TIF) [file pgen.1005858.s005.tif]
